# Osseointegrated Percutaneous Prosthetic System for the Treatment of Patients With Transfemoral Amputation: A Prospective Five-year Follow-up of Patient-reported Outcomes and Complications

**DOI:** 10.5435/JAAOS-D-17-00621

**Published:** 2018-12-13

**Authors:** Rickard P. Brånemark, Kerstin Hagberg, Katarzyna Kulbacka-Ortiz, Örjan Berlin, Björn Rydevik

**Affiliations:** From the Centre for Advanced Reconstruction of Extremities (C.A.R.E.), Sahlgrenska University Hospital, Department of Orthopaedics, Institute for Clinical Sciences, The Sahlgrenska Academy, University of Gothenburg, Mölndal, Sweden (Dr. Brånemark, Dr. Hagberg, Mrs. Kulbacka-Ortiz, and Dr. Berlin), International Centre for Osseointegration Research, Education and Surgery (iCORES), Department of Orthopaedics, University of California, San Francisco, CA (Dr. Brånemark), and the Sahlgrenska University Hospital, Department of Orthopaedics, Institute for Clinical Sciences, The Sahlgrenska Academy, University of Gothenburg, Mölndal, Sweden (Dr. Rydevik).

## Abstract

Direct skeletal attachment of prostheses has previously been shown to improve patient-reported outcome (PRO) measures of individuals with transfemoral amputation (TFA) at 2-year follow-up. This prospective study reports the outcomes at 5-year follow-up.

**Methods:**

A total of 51 patients (55 legs) with TFA were included in a prospective study. Complications, success rate, and PRO measures were followed for 5 years.

**Results:**

The cumulative fixture survival rate at 5 years was 92%, and the revision-free survival rate was 45%. Thirty-four patients had 70 superficial infections. Eleven patients had 14 deep infections. Fifteen patients had mechanical complications. Four fixtures were removed (ie, one deep infection and three loosening). PRO measures showed significant improvements including more use of the prosthesis, better mobility, fewer issues, and improved physical health-related quality of life (all *P* < 0.0001) compared with baseline.

**Conclusion:**

Individuals with TFA at 5-year follow-up had significant improvement in PRO measures, but increases in deep infections and mechanical complications are concerning.

Lower limb amputees frequently experience issues related to socket prostheses. Chafing, pain, skin sores, and perspiration issues can occur in up to 70% of patients with transfemoral amputations (TFAs) due to nondysvascular conditions such as trauma or tumor, and these socket-related issues do not seem to have been substantially improved during the past 30 years, despite improvements in the socket design.^1-3^ The fundamental issue related to load transfer from the body via the soft tissues to the socket and then to the prosthesis does not seem to be easily resolved. In the late 1960s, attempts were made by Mooney et al^4^ to overcome these issues by using percutaneous bone anchored implants. However, neither long-lasting anchorage to the bone nor infection control was achieved. The treatment presented in this article is based on the concept of osseointegration, which was developed by Brånemark et al^5^ and revolutionized the treatment of edentulousness.^6,7^ Further successful applications were developed for bone-anchored hearing aids,^8^ craniofacial prostheses,^9^ and thumb amputation prostheses.^10^ Starting in 1990, TFA patients were treated with custom-designed implants and varying surgical techniques and nonstandardized rehabilitation procedures. Since 1998, standardized implants, surgical instruments, surgical techniques, and rehabilitation procedures have been used. Despite the good results on a short-term basis of the OPRA (Osseointegrated Prostheses for the Rehabilitation of Amputees) study,^11^ the technique still remains controversial. Other authors have reported similar good short-term results but with a different concept of bone anchorage.^12,13^ The main concerns relate to mechanical longevity of the implant system, durability of the bone anchorage, rate of infection, and whether the reported improvement of patient-reported outcome (PRO) measures will stand the test of time. The purpose of this article was to report the prospective 5-year follow-up results of PRO measures compared with the patients' status at treatment start, complications, and success rate.

## Methods

This was a prospective, single-center, nonrandomized study in accordance with the European standard for clinical investigations of medical devices (EN-540) for which ethical approval had been obtained (R402-98). All patients signed a written consent form. Patients were recruited according to the following inclusion criteria: transfemoral amputees (age 20 to 70 years) with issues related to conventional socket-suspended prostheses or unable to use a prosthesis or not using a prosthesis, and probably complying with the treatment and follow-up requirements as judged by the treatment team consisting of experienced orthopaedic surgeons, physiotherapists, and prosthetists. The exclusion criteria were amputation due to severe peripheral vascular disease and/or diabetes mellitus, skin diseases on the amputated limb, pregnancy, and current treatment with systemic corticosteroids, chemotherapeutic agents, or other drugs that could adversely affect the treatment. Pre- and post-operative data regarding the osseointegrated prostheses were compared for each patient at baseline and at follow-up. A total of 51 patients with TFA were included in the study between 1999 and 2007, and each patient was followed up for 5 years.

**Figure 1:**
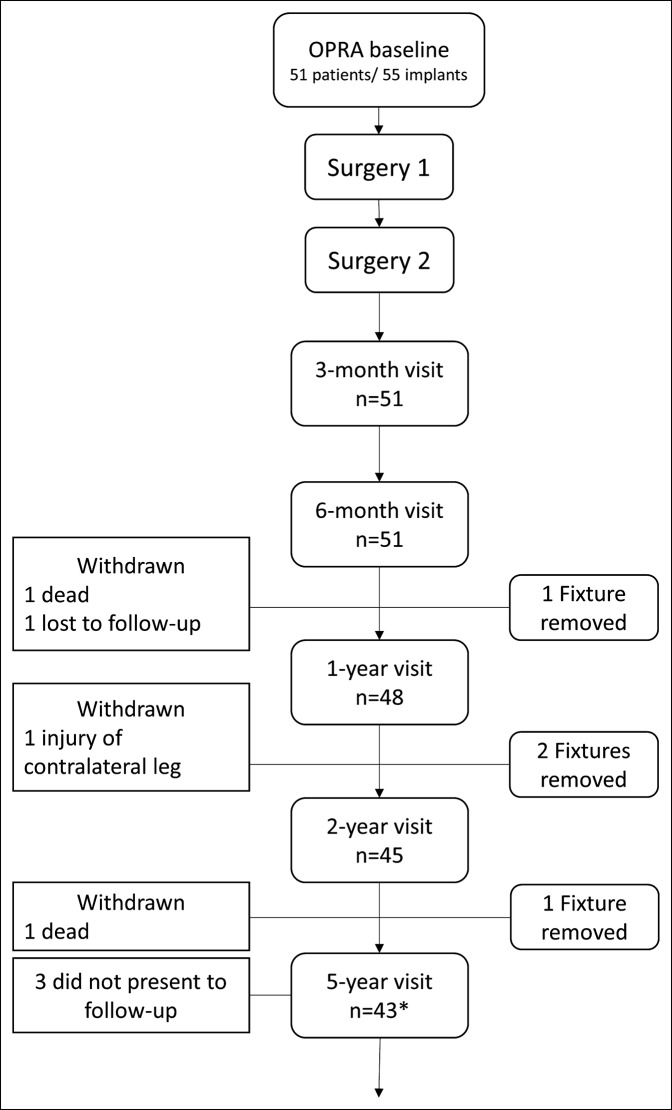
Flowchart showing patient participation and patients lost to follow-up in the study over time. *At 5-year follow-up, 40 patients of 43 possible were followed.

Forty-five patients had unilateral and six bilateral TFAs (Table 1, Supplemental Digital Content 1, http://links.lww.com/JAAOS/A297). Four of the six patients with bilateral TFAs were treated bilaterally, whereas two with bilateral TFAs were treated on one side only. Thus, the total number of limbs evaluated was 55. The main reasons for amputation were trauma and tumor. At inclusion, 42/51 patients were using socket-suspended prostheses, but nine did not use any prostheses because of difficulty obtaining adequate socket fit. Among those, eight had tried to use a prosthesis, and one had not. All surgeries were performed at the Sahlgrenska University Hospital, Gothenburg, Sweden. The design of the study and loss to follow-up are presented in Figure 1.

### Treatment Protocol

**Figure 2:**
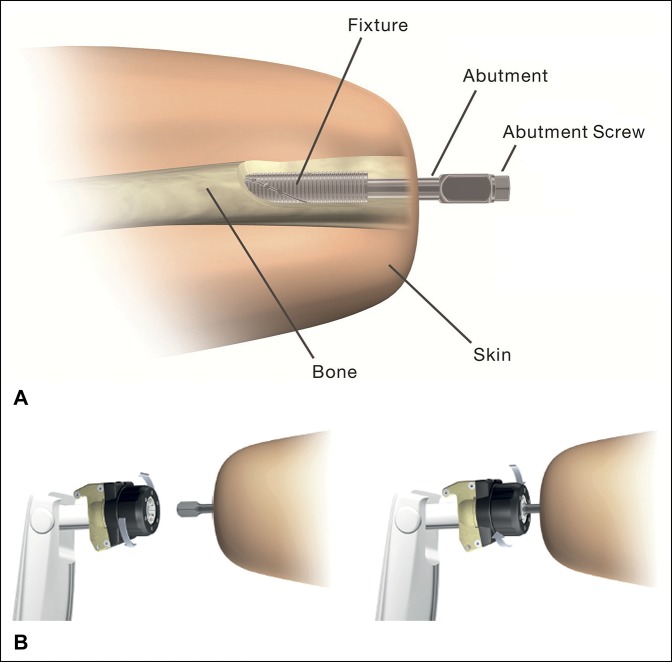
**A**, Schematic showing the implant system. The system contains three main components: fixture, abutment, and abutment screw. **B**, Schematic showing the attachment device connecting the implant to the external prosthesis. (Part A reprinted with permission from Brånemark R, Berlin Ö, Hagberg K, Bergh P, Gunterberg B, Rydevik B: A novel osseointegrated percutaneous prosthetic system for the treatment of patients with transfemoral amputation: A prospective study of 51 patients. *Bone Joint J* 2014;96-B:106-113.)

The implant system (OPRA Implant System, Integrum AB, Mölndal, Sweden) consists of three main components: the fixture, providing bone anchorage, the abutment connected to the fixture by press fit and by the retention of the abutment screw, and the distal end of the abutment is connected to an additional safety device (with a release mechanism in torsion) and then to the external prosthesis (Figure 2, A and B). The fixture is protected from mechanical overload in bending by using the abutment as mechanical fuse; the modular design makes it possible to replace the abutment if bent or fractured as a day surgery procedure. Hence, removal of the fixture was considered the end point for failure in the clinical investigation plan.

The treatment involves two surgeries (ie, S1 and S2) separated by 6 months, followed by rehabilitation. At S1, the intramedullary canal is reamed to the appropriate size, and the fixture is installed, using a three-dimensional centering guide and fluoroscopy to ensure correct positioning. The fixture is left unloaded in the bone during the healing period to enable it to be osseointegrated.^5^ At S2, all distal muscles are guillotined and sutured to the periosteum, leaving the bare bone protruding by approximately 5 mm, covered only by part of the skin flap, which is trimmed of subcutaneous fat to full skin thickness and attached to the end of the bone.^11,14^ The abutment is inserted through the skin, into the fixture, and secured with the abutment screw. Rehabilitation after S2 entails gradually increasing both the load on the implant and prosthetic use and activity.^15^

At predefined follow-ups (ie, 3, 6, 12, and 24 months after S2), a clinical examination and safety assessment were made. Any complication, registered as an adverse event (AE; event which is easily tolerated or which causes sufficient discomfort to interfere with daily activities) or serious AE (as a result of an AE during a clinical investigation, where a patient has to be hospitalized, or their hospitalization is unduly prolonged because of potential disability or danger to life or because an intervention has been necessitated or the event is terminal), was recorded on case record forms (CRFs). For example, the skin penetration area was assessed for redness and secretion. If there were signs of superficial or deep infection, additional information on infection history and treatment had to be entered on the CRF. At 5-year follow-up, a clinical assessment was made, radiographs were performed, and PROs were recorded. In addition, any AEs directly related to the treatment were noted in the medical records. If the patient had been assessed by another caregiver, copies of their medical records were reviewed by one of the study investigators.

### Patient-reported Outcome Measures

Efficacy variables were captured using two valid, reliable questionnaires: the Questionnaire for persons with Transfemoral Amputation (Q-TFA)^16^ and the Short Form 36 (SF-36) Health Survey.^17^ Both were answered before S1 and 1, 2, and 5 years after S2.

The Q-TFA presents the results as four scores: Prosthetic Use Score (0 to 100), Prosthetic Mobility Score (0 to 100), Problem Score (100 to 0, note this score is reversed), and Global Score (0 to 100). A Prosthetic Use Score of 0 means that the prosthesis is not used at all, whereas 100 means that the prosthesis is used 7 d/wk for >15 hr/d. In addition, two osseointegration-specific questions were included at 2- and 5-year follow-ups regarding the perceived degree of issues with the skin penetration area and the perceived degree of being worried about complications with the prosthetic anchorage during the past 3 months, respectively.

The SF-36 is a general health-related quality of life (HRQL) questionnaire resulting in eight subscales and two summary measures each giving a score of between 0 and 100, with a higher figure representing better HRQL.^17,18^ The summary measures, the Physical and Mental Component Scores, are standardized to the general population.

The primary outcome variable is the Prosthetic Use Score. The primary safety variable is the fixture cumulative survival rate. Mechanical complications (ie, abutment and abutment screws) are secondary outcome variables.

### Statistical Methods

All data, including any AEs, were collected on CRFs up to 2 years and then in the medical records. Statistical analyses were performed with the SAS version 9.2 (Statistical Analysis System) in accordance with a prespecified statistical plan for the 2-year data, and thereafter, SPSS Statistics version 21 (IBM) was used. Descriptive statistics were used for the number of patients, mean, SD, median, minimum and maximum values, and frequencies and percentages for categoric variables. Fixture survival was calculated using the cumulative success rate. The Wilcoxon signed-rank nonparametric test and the sign test were used to analyze within-group differences. The Mann-Whitney *U* test was used to analyze differences between the groups. The significance tests were performed at subject level and were two sided and conducted at the 5% significance level.

## Results

**Figure 3:**
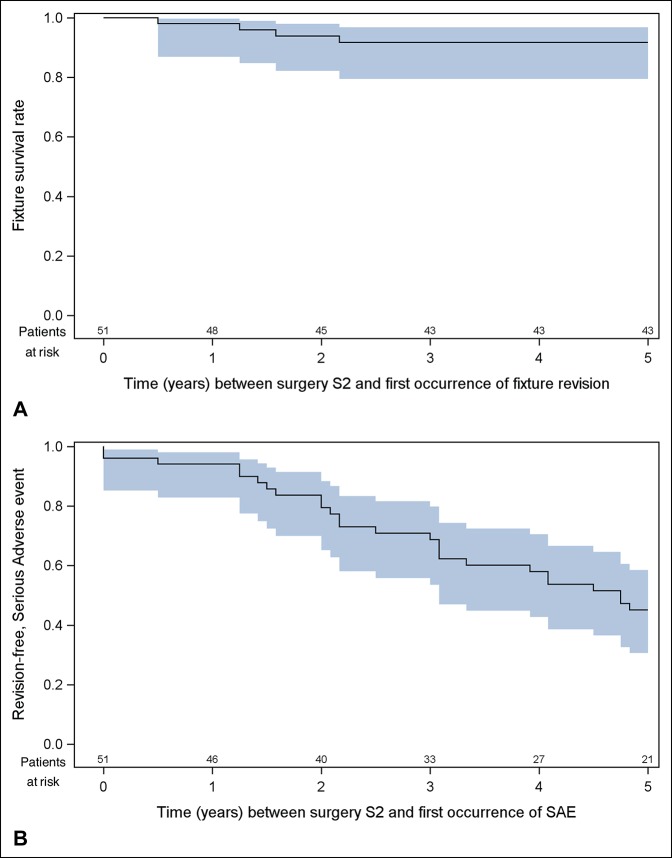
**A**, Kaplan-Meier graph showing survival of the fixture over time. **B**, Kaplan-Meier graph showing the revision-free rate over time. SAE = serious adverse event

Forty-five of 51 patients passed 2-year follow-up; three patients were withdrawn from the study for reasons unrelated to the implant (ie, one death from an unrelated cause, one severe dysfunction of the contralateral knee, and one lost to follow-up). Three patients had their implants removed within 2 years and one shortly after, and this failure was included in the 2-year results.^11^ A total of 40 patients were followed up at 5 years (Figure 1). At this time point, one additional patient was withdrawn from the study (deceased for an unrelated reason at 3-year follow-up), and three patients did not present for 5-year follow-up (ie, one patient living in Spain, who showed up at 7-year follow-up with no issues, and two patients with psychiatric disorders, both with their implants in place). The 5-year fixture cumulative survival rate was 92% (Figure 3, A), and the revision-free rate was 45% (Figure 3, B).

The most common AE was superficial infection, occurring 70 times in 34 patients from baseline to 5-year follow-up. Superficial skin infections were normally treated with oral antibiotics for 10 days, but 16 of them required longer treatment. A total of 85 serious AEs were reported in 26 patients (ie, removal of the fixture [4 patients], stump revisions [3 patients], deep infections [11 patients], and exchange of the abutment and/or abutment screw [15 patients]). A total of 14 deep infections were diagnosed in 11 patients during the 5-year period. One of these infections caused early loosening/failure of the fixture. Nine patients with deep infections were successfully treated with oral antibiotics, with a mean time of 5 months. One deep infection had not resolved at 5-year follow-up (Table 2, Supplemental Digital Content 2, http://links.lww.com/JAAOS/A298).

Forty-three mechanical complications occurred in 15 patients, resulting in replacement of the damaged abutment and/or the abutment screw. Accidental overload (ie, falling and stumbling causing the abutment to bend) was the cause in 16 bent abutments in 9 patients. One patient had the abutment temporarily removed (fixture in situ) four months before the 5-year follow-up appointment because of mechanical issues with the abutment and abutment screw. All reported AEs up to 5-year follow-up are presented in Table 3 (Supplemental Digital Content 3, http://links.lww.com/JAAOS/A299).

**Figure 4:**
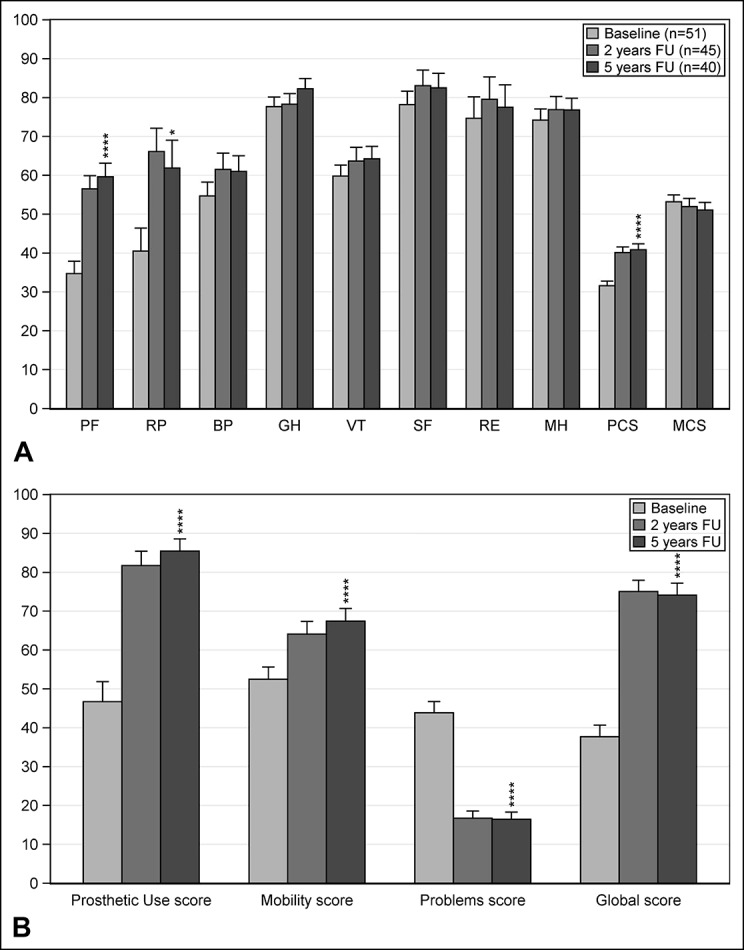
**A**, Graph showing results of the Short Form 36 at baseline, 2-year, and 5-year follow-up. BP = Bodily Pain, GH = General Health, MCS = Mental Component Score, MH = Mental Health, PCS = Physical Component Score, PF = Physical Functioning, RE = Role Emotional, RP = Role Physical, SF = Social Function, VT = Vitality. The PCS and MCS are normed to mean 50 and SD 10^18^; all other scores 0 to 100. *RP, PCS and MCS n = 50 due to one missing in the RP score; *P < 0.05, and ****P < 0.0001. Error bars represent mean + standard error of the mean (SEM). Analyses of differences between baseline and 5-year follow-up showed statistically significant improvement in the PF score (*P* < 0.0001), RP score (*P* = 0.020), and PCS (*P* < 0.0001), all other differences nonsignificant (ns). Analyses of differences between 2-year and 5-year follow-up were all ns. Analyses of differences between baseline and 2-year follow-up previously reported^11^ and showed statistically significant improvement in the PF score, RP score, and PCS (*P* < 0.001), all other differences ns. **B**, Results of Q-TFA at baseline, 2-year, and 5-year follow-up; *P < 0.05, and ****P < 0.0001. Error bars represent mean + standard error of the mean (SEM). Number of patients included at baseline, 2-year, and 5-year follow-up were 51, 44, and 40 in the Prosthetic Use Score and 42, 44, and 40 in all other scores, respectively. All Q-TFA scores are 0 to 100, but for the Problem Score, which is reversed (100-0). Values of Q-TFA Mobility, Problem, and Global Scores cannot be calculated if the Prosthetic Use Score is 0^16^. At baseline, 9 patients reported not using a prosthesis (Prosthetic Use Score = 0). Analyses of differences between Baseline and 5-year follow-up showed statistically significant improvements in all four Q-TFA scores (*P* < 0.0001). Analyses of differences between 2-year and 5-year follow-up were all ns. Analyses of differences between baseline and 2-year follow-up previously reported^11^ and showed statistically significant improvements in all four Q-TFA scores (*P* < 0.001).

**Figure 5:**
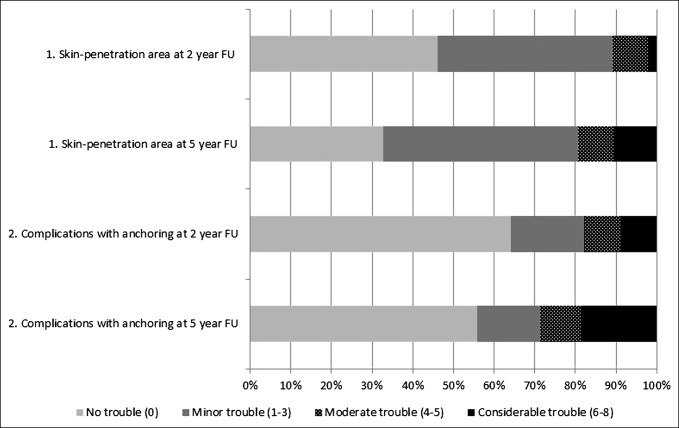
Percentage of patients reporting osseointegration-specific issues with regard to the skin penetration area and worries about prosthetic anchorage at 2- and 5-year follow-up, respectively. FU = follow-up. Number of patients answering the questions were at 2-year follow-up n = 44 and at 5-year follow-up n = 39. There was one missing at each follow-up. The wording and grading of the two questions were as follows: 1a. During the past 3 months, have you experienced irritation/infection in/around the skin penetration area? (0 = no trouble to 4 = great deal of trouble) and 1b. How has this affected your quality of life (QL)? (0 = no reduction in QL to 4 = extreme reduction in QL)*.* 2a. During the past 3 months, have you been worried about the complications with the prosthetic anchoring? (0 to 4) and 2b. How has this affected your quality of life? (0 to 4). Combining the answers in a + b for each question (1a + 1b and 2a + 2b) gives a figure between 0 and 8. The percentage of patients reporting 0 = no trouble, 1 to 3 = minor trouble, 4 to 5 = moderate trouble, and 6 to 8 = considerable trouble are reported. For analyses of differences between 2-year and 5-year follow-up, the related sample sign test was used. The results showed nonsignificant differences in both questions: skin penetration area (*P* = 0.48) and worries about the prosthetic anchoring (*P* = 0.48).

Efficacy data according to the Q-TFA and SF-36 are presented in Figure 4, A and B and Table 4 (Supplemental Digital Content 4, http://links.lww.com/JAAOS/A300). Analyses of differences between baseline and 5-year follow-up revealed statistically significant improvements in all four Q-TFA scores (*P* < 0.0001) and in the Physical Function (*P* < 0.0001), Role Physical (*P* = 0.020) and Physical Component scores (*P* < 0.0001) on the SF-36. All other differences were nonsignificant. Details of prosthetic use at baseline showed that 29/42 (69%) used their prostheses on a daily basis for at least 13 hr/d. At 5-year follow-up, this was reported by 28/40 (70%) patients. The results from the two osseointegration-specific questions are reported in Figure 5, and no statistically significant differences between the 2- and 5-year follow-up periods were found.

To address a possible relation between higher prosthetic activity and mechanical complications, the group of 40 patients at 5-year follow-up was divided into those that had experienced any mechanical complication to the abutment and/or abutment screw and those without any such complication and compared with regard to their Q-TFA Mobility Score at 5-year follow-up. The result showed a statistically significant higher mean Mobility Score in the group of patients that had had a mechanical complication (*P* = 0.035). The group with any mechanical complication (n = 15) had a Mobility Score of 74 (SD, 20.5; 8 to 92) compared with the group without any complication (n = 25), which had a Mobility Score of 64 (SD, 20.2; 4 to 97).

## Discussion

In comparison with the previous report of the OPRA study with only short-term follow-up (2 years),^11^ the aim of this study was to see whether the promising results prevailed with a mid-term perspective (5 years). Regarding the longevity of the treatment, no additional patient lost the anchorage (fixture) of the implant system. Mechanical complications increased and were more frequent in patients with higher activity. Deep infections increased, but superficial infections per patient and year remained constant. The PRO measures at 5-year follow-up were similar to those at 2-year follow-up.

The use of bone-anchored devices for the treatment of individuals with amputations has more than 25 years of clinical experience, but there are still many important questions to address, such as long-term success and complications. In this early phase, it is critical to follow well-accepted guidelines for the implementation of new medical devices, as described by Malchau.^19^ After initial pilot studies, longer controlled prospective studies including tools to predict implant migration should be followed by multicenter studies.^19^ A prospective study using another implant system for bone anchorage^20^ reported similar improvement in PROs. The use of radiostereometeric analysis^21^ showed no significant implant migration after 2-year follow up, and the present report showed no “late” fixture loosening, which suggest that RSA can be used to predict long-term function, not only for hip stems but also for the studied percutaneous osseointegrated system. In a recent review,^22^ it has been suggested that patients should be followed up long term, and, to date, this 5-year report is the longest prospective follow-up study of AEs, survival data, and PROs in relation to percutaneous orthopaedic osseointegrated implants.

Other attempts at percutaneous bone-anchored prostheses have recently been published with promising results in TFA, in a prospective case series of 22 Dutch patients referenced earlier,^20^ a retrospective series of 69 German patients,^12^ and case studies.^23,24^ The concept has also been used in individuals with transhumeral amputations, presented as a case series in 18 patients^25,26^ and a case study.^27^ Potentially important differences exist between the implant systems used. Aschoff and Juhnke^28^ have developed a design using a femoral stem with a meshed surface with no threads. This concept has further been developed by Al Muderis with the so-called Osseointegration Group of Australia Accelerated Protocol system, which suggests a speeded protocol with a one-stage procedure,^29^ which has benefits in terms of treatment time, but also adds risks of implant loosening.^30^ A more thorough review on the design of the different systems presently used or in development was recently published by Thesleff et al.^31^

The 2-year follow-up period reported more prosthetic use, better mobility, fewer issues, improved overall situation, and improved general physical HRQL (all *P* < 0.001) with the use of osseointegrated prostheses compared with the patients' previous situation.^11,32^ The 5-year data remain stable with very similar improvements, as shown in Figure 4, A and B and Table 4 (Supplemental Digital Content 4, http://links.lww.com/JAAOS/A300). At 5-year follow-up, no additional fixture removals exist, which is encouraging. The benefits are regarded as clinically relevant and are supported by the statistical evaluations. The efficacy of the treatment is also supported by a published in-depth qualitative study^33^ comprising 13 patients fitted with osseointegrated amputation prostheses describing the “revolutionary change” the treatment has produced in their lives.

Superficial infections remain at about one per patient every second year up to 5 years, and our experience points toward regarding this as a minor complication with only a limited impact on everyday life. Deep infections, however, constitute a major complication that, despite long-term treatment with single or dual antibiotics, sometimes requires surgical interventions. It is somewhat encouraging that, despite deep infections, only one implant had to be removed because of infection, and this was an early deep infection before successful osseointegration had been established. This phenomenon corroborates previous studies showing the “durability” of the osseointegrated interface,^34^ as well as several clinical reports on well-fixed endoprostheses.^35^

One patient with a recurrent infection of the distal bone, but no signs of proximal osteomyelitis, was treated with antibiotics at 5-year follow-up. Swabs taken from the skin penetration site 1 month later revealed a superficial infection (*Staphylococcus aureus* and coagulase-negative *Staphylococcus*). The patient received additional antibiotic treatment with clindamycin for 2 months. One patient had the abutment temporarily removed. This patient was an active prosthetic user and had six of the total of nine reported mechanical events at 2-year follow-up. The patient then had four additional abutment and abutment screw changes and one abutment screw changed separately, possibly initiated by overloading the system and bending the abutment. In all, this patient had eight abutments and abutment screws changed and three abutment screws changed separately. Eleven months later, at his 6-year follow-up, the patient decided to have an abutment reinstalled. Overload of the implant system due, for example, to a fall can result in the bending of the abutment and possibly also some deformation of the connection part of the fixture. Between the 2- and 5-year follow-up periods, eight patients had bent abutments.

The patients' general perception of the number of complications is also visible in their answers relating to specific issues perceived in conjunction with the osseointegration treatment. As shown in Figure 5, the percentage of patients reporting these issues is somewhat higher at 5-year follow-up compared with 2-year follow-up. However, the vast majority of patients report minor or no trouble.

There are limitations to this study, mainly that the number of patients was small and that four patients were withdrawn from the study and three patients were unavailable for 5-year follow-up. The study was not randomized for obvious reasons, nor was it multicenter, and the follow-up period was only mid-term (5 years). Finally, the two osseointegration-specific questions described in Figure 5 have not been validated or tested for reliability and responsiveness.

As shown in the Results section, 70% of the patients reported using their prosthesis every day ≥13 hr/d at follow-up. This can be regarded as very high use of TFA prostheses compared with the published literature on socket-suspended prostheses,^32,36^ especially as the study group includes patients with short residual limbs, other functional limitations, and bilateral amputations (Table 1, Supplemental Digital Content 1, http://links.lww.com/JAAOS/A297). The current result showed a higher Q-TFA Mobility Score among those that had had mechanical complications than for those with no complications. However, as shown in the Results section, both groups include patients with low to high Mobility Scores. Further research regarding mechanical complications and their possible relations to prosthetic activity should be included in future investigation, potentially including step counters, gait analyses, and load cells to characterize the loads and frequency.

The 5-year follow-up period shows a continuous cumulative fixture (bone anchorage) survival rate of 92%, but the revision-free rate for any cause is 45% because of an increased number of mechanical complications of the abutment and abutment screw and an increase in deep infections, which are concerning. Four patients have had the entire implant system removed. The incidence of superficial infections remains at one per patient per every second year. The PRO results are significantly improved compared with baseline and remain stable from a short- (2-year) to a mid-term (5-year) follow-up period.

## Conclusion

Individuals with TFA at 5-year follow-up had significant improvement in PROs, but increases in deep infections and mechanical complications are of concern.
